# Combined Analysis with Copy Number Variation Identifies Risk Loci in Lung Cancer

**DOI:** 10.1155/2014/469103

**Published:** 2014-07-01

**Authors:** Xinlei Li, Xianfeng Chen, Guohong Hu, Yang Liu, Zhenguo Zhang, Ping Wang, You Zhou, Xianfu Yi, Jie Zhang, Yufei Zhu, Zejun Wei, Fei Yuan, Guoping Zhao, Jun Zhu, Landian Hu, Xiangyin Kong

**Affiliations:** ^1^Institute of Health Sciences, Shanghai Jiao Tong University School of Medicine (SJTUSM) and Shanghai Institutes for Biological Sciences (SIBS), Chinese Academy of Sciences (CAS), Shanghai 200025, China; ^2^Chinese National Human Genome Center at Shanghai, Shanghai 201203, China; ^3^Institute of Bioinformatics, College of Agriculture and Biotechnology, Zhejiang University, Hangzhou 310029, China; ^4^State Key Laboratory of Medical Genomics, Ruijin Hospital, Shanghai Jiao Tong University, 197 Ruijin Road II, Shanghai 200025, China

## Abstract

*Background*. Lung cancer is the most important cause of cancer mortality worldwide, but the underlying mechanisms of this disease are not fully understood. Copy number variations (CNVs) are promising genetic variations to study because of their potential effects on cancer. *Methodology/Principal Findings*. Here we conducted a pilot study in which we systematically analyzed the association of CNVs in two lung cancer datasets: the Environment And Genetics in Lung cancer Etiology (EAGLE) and the Prostate, Lung, Colorectal and Ovarian (PLCO) Cancer Screening Trial datasets. We used a preestablished association method to test the datasets separately and conducted a combined analysis to test the association accordance between the two datasets. Finally, we identified 167 risk SNP loci and 22 CNVs associated with lung cancer and linked them with recombination hotspots. Functional annotation and biological relevance analyses implied that some of our predicted risk loci were supported by other studies and might be potential candidate loci for lung cancer studies. *Conclusions/Significance*. Our results further emphasized the importance of copy number variations in cancer and might be a valuable complement to current genome-wide association studies on cancer.

## 1. Introduction

Lung cancer is the most common cause of cancer-related death in the world [1]. Many types of genetic variation such as single-nucleotide polymorphisms (SNPs) and copy number variations (CNVs) [2–7] have been discovered to be associated with lung cancer. Copy number variations are prevalent in the genome, covering approximately 12% of the human genome [8], which may make it more likely to contribute to disease incidence [9–15]. Thus, a systematic survey of CNVs in lung cancer is essential.

To test the relationship between copy number variations and lung cancer susceptibility, we conducted a pilot combined genome-wide analysis of two case-control datasets in lung cancer, consisting of 1,945 cases and 1,992 controls from the Environment And Genetics in Lung cancer Etiology (EAGLE) project [16] and 803 cases and 848 controls from the Prostate, Lung, Colon and Ovary (PLCO) Study Cancer Screening Trial project [17]. All cases and controls were matched well for age, gender, district, and other characteristics according to the original study design. Genomic DNA samples from somatic cells (blood cells) were probed using an Illumina HumanHap 550K genotyping chip. Before testing, we normalized all data (including a training dataset of 66 individuals from HapMap) using quantile normalization. To infer the copy number state of each SNP site, we trained a well-established hidden Markov model (HMM) using the training dataset. All of the above procedures were performed as previously described [18]. Our combined association analysis was done at two levels. First, in each individual dataset we conducted an SNP-based testing to probe the association of lung cancer with a specific SNP site. Next, window-based testing was performed to probe the association pattern with lung cancer. The details of the SNP-based and window-based testing are in Section 4. Second, to test the association accordance between the two datasets, we conducted a combined analysis in which a new statistic of relative factor (Rf) was calculated for each SNP site. In the calculation of the Rf, the cases and controls from different datasets were hypothesized to have an independent genomic distribution of copy number states. We assumed that the difference between the two copy number state distributions could be tested from their accordance and the combination of related *P* values could be used to depict such difference between these two datasets (see details in Section 4).

## 2. Results

### 2.1. Raw CNVs Prediction for EAGLE and PLCO

We used the full set of study participants as described in Section 4. After quality control of the samples and array data, the probe signals on the chip were transformed to copy number state by our preestablished HMM approach [18]. We first adopted a simple CNV calculation method to roughly generate raw CNVs for both the EAGLE and PLCO datasets (see Section 4). We compared the raw CNVs between EAGLE and PLCO (Table 1) and found that although the average span and size of raw CNVs were comparable between EAGLE and PLCO, the number of raw CNVs predicted by this approach was larger in EAGLE than in PLCO. This might be caused by the smaller sample size of PLCO used in this study than EAGLE when studying potential rare risk loci. Meanwhile, as this simple raw prediction method was based only on individual level copy number state data, false positive noises in each individual might also increase the total number of predicted CNVs when sample size increased. Given these considerations, we assumed that the simple raw CNV calculation method we used might not be appropriate to predict reliable CNVs between different datasets. As a result, we developed another combined strategy following our preestablished CNV association approach to predict CNVs between EAGLE and PLCO. This combined strategy was based on the copy number state distributions in both EAGLE and PLCO individuals, which might help to filter out false positive noises in single individual level prediction. Moreover, since the whole copy number state distributions of both EAGLE and PLCO cohorts were considered and integrated, CNVs predicted in this way were not dependent on single cohorts, which we expected to avoid the impact of different sample sizes on CNVs prediction (see Section 4 for details).

### 2.2. Genome-Wide Combined Analysis for EAGLE and PLCO

**Figure 1:**
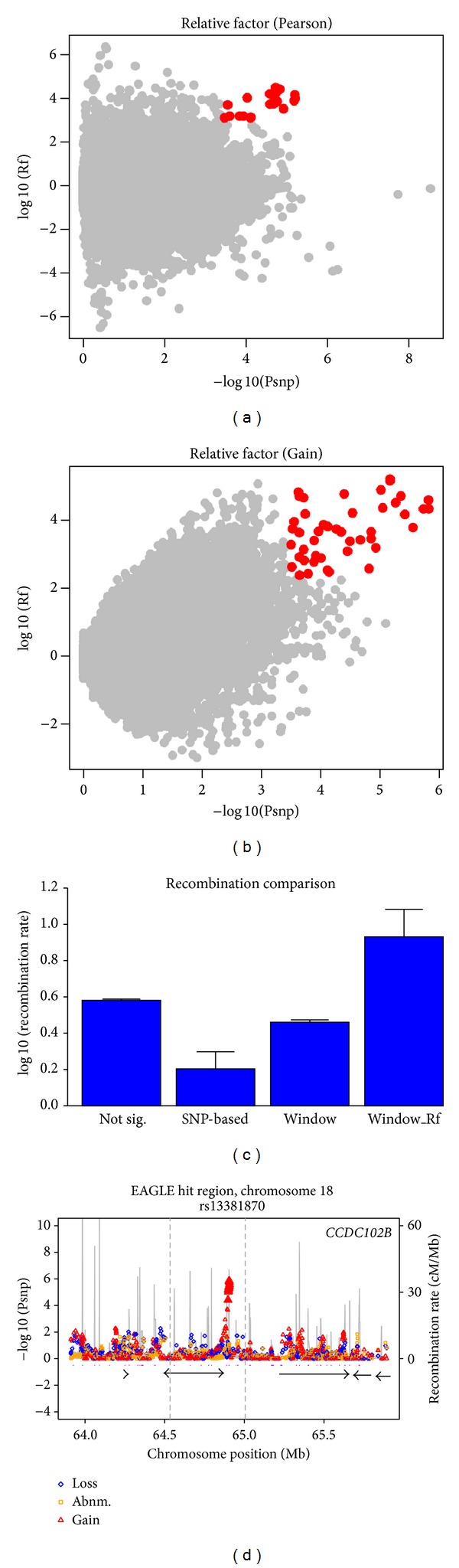
CNV associations regarding amplification are related to recombination hotspot regions in EAGLE and PLCO. The −log 10 (*P* value) in the (a) Pearson testing and (b) Gain testing are plotted against the corresponding log 10 (relative factors). The SNP sites above a significant level (combining *P* value and relative factor to ensure that the final false positive is less than 1) are in red. (c) The log 10 (maximum recombination rate) around the SNP sites (in 10 kb) are summed in four categories: not sig. (nonsignificant SNP sites), SNP-based (significant SNP sites that passed SNP-based testing), window (significant SNP sites that passed window-based testing), and window_Rf (risk loci that passed both window-based and combined testing). The log 10 (maximum recombination rate) is prevalent in the category of window_Rf (*P* value < 0.00001). (d) Most of these risk loci were located around recombination hotspots (plotted in gray lines and with peaks indicating the recombination rates). One of these associated sites, rs13381870, was arbitrarily chosen and is shown here as an example. The –log 10 (*P* value) of SNP sites in three hypotheses models (*loss*,* abnm.*, and* gain*) in SNP-based testing are plotted in blue, orange, and red, respectively. Grey vertical lines show the high recombination rate sites. Hotspots from HapMap were shown as purple bars between the plot and genes. The names of genes around rs13381870 are shown in the figure.

In our previous study [18], we developed a two-step genome-wide CNV association approach based on SNP-based testing and window-based testing to find significant SNP sites with abnormal copy number variations. Here we used the same approach as the initial stage of our combined analysis. In the SNP-based testing, we obtained 509 candidate SNP sites in EAGLE and 573 candidate SNP sites in PLCO (the corresponding FDR ≤ 0.15) (Figure S1, available online at http://dx.doi.org/10.1155/2014/469103). We noticed that more SNP candidates were found in PLCO than in EAGLE; the reason might be that larger sample size could help reduce false positive noises in SNP-based testing. All SNP candidates were subjected to window-based testing after SNP-based testing. As expected, we found that statistical power in PLCO was lower than in EAGLE (Figure S2). The reason for this phenomenon might be that PLCO contained a smaller sample size than EAGLE, as a loss of statistical power in small sample sizes had been recognized in other studies [19, 20]. Therefore, in our subsequent analyses EAGLE was used as the discovery dataset and PLCO was used to verify the association accordance between them. In the EAGLE dataset, we identified 355 SNP sites with significant window-based *P* values (FDR = 0.0702482) (Table S1). In the PLCO dataset, 243 SNP sites passed window-based testing (FDR = 0.102871). Genome-wide association testing in a single dataset might be influenced by population structure and other factors, leading to false positives in many studies [21, 22]. Indeed we observed such population stratification in the EAGLE dataset, although there was no obvious stratification between case and control cohorts (Figure S3). Therefore, a strict combined analysis integrating PLCO with EAGLE was conducted to evaluate the association results in EAGLE. After the combined analysis, 167 SNP sites were obtained as risk loci in EAGLE (Table S2) and good association consistency between EAGLE and PLCO datasets was found in the hypothesis regarding amplification (Figures 1(a) and 1(b)). For the other two hypotheses regarding deletion and abnormal (deletion or amplification), the association consistency was not as good as that of amplification (data not shown), which might be caused by the population differences between EAGLE and PLCO or the limitations of our approaches.

### 2.3. Functional Annotation Clustering Analysis of Genes around Risk Loci

To study the biological meaning of those significant risk sites in EAGLE, we did a functional annotation clustering analysis of genes surrounding those sites. Since there was no more evidence to show which gene will be affected by the candidate risk loci, we roughly glanced at genes located within ±100 kb around those risk loci and retrieved a list of 243 neighboring genes (Table S3) of all the risk loci in Table S2.

Next, we used DAVID (http://david.abcc.ncifcrf.gov/) for a functional clustering analysis of the neighboring gene list to see whether there were some biologically meaningful clusters. From the results we found that there were some gene sets formed in annotation cluster 4 (see Table S4 for detailed and statistical information) as defined by SMART and INTERPRO classification. We found that in spite of the many gene sets in annotation cluster 4, many of them did not have a significant statistical *P* value. We assumed two reasons for this phenomenon. First, our definition of affected genes was arbitrary which might lead to the inclusion of unrelated background genes or exclusion of truly affected genes, and both situations would lower the clustering power of DAVID. Second, the underlying mechanisms of a complex disease like lung cancer cannot be easily modeled by such a simple study approach and as a result the less significant clustering sets might only reveal a small piece of the whole network.

In DAVID analysis, we compared our neighboring gene list with the published literature. We selected the PubMed ID for DAVID to see which subset of genes was related to previous studies. Two published studies were reported to be significantly related to neighboring gene list (Table S5). PMID 11085536 [23] was a study that included twelve of the genes in neighboring gene list. The authors of that study tested manually and found loss of expression or reduced mRNA levels for* SEMA3B* in both small cell and non-small cell lung cancers, as well as reduced mRNA levels of* CACNA2D2* in non-small cell lung cancer and two or more sequence-altering mutations for* SEMA3B* and* NPRL2*, indicating that those genes might be candidate tumor suppress genes (TSGs). The study in PMID 19140316 [24] found four genes also in our neighboring gene list. They used real-time PCR to analyze the downregulation of four genes,* HYAL1*,* HYAL2*,* RASSF1A*, and* NPRL2*, in lung cancer and found that they were downregulated in non-small cell lung cancer, the first stage of squamous cell lung cancer, and were significantly associated with lung adenocarcinoma progression. They expected the downregulation of those genes to be important for diagnosis and therapeutic strategies development of lung cancer. The fact that our neighboring gene list also contained those previously reported genes revealed that genes around the 167 risk loci were worthy of future functional studies.

### 2.4. CNVs around Risk Loci and Their Biological Relevance

We carefully investigated the 167 risk loci in EAGLE that passed SNP-based, window-based, and combined analysis with PLCO (Table S2) and found that those risk loci could be classified into two groups depending on their consecutiveness: singular risk loci, which were short of flanking risk loci, and consecutive risk loci, which consisted of many consecutive flanking risk loci, forming a CNV risk region. Based on this classification, we manually checked all 167 risk loci and generated CNVs from the consecutive risk loci blocks (Table 2; see Section 4).

A total of 22 CNVs were summarized from 167 risk loci in EAGLE, including three amplification CNVs, 18 deletions, and 1 abnormal (amplification/deletion) variation. As we mentioned previously, our combined analysis had a good association consistency regarding amplification (Figures 1(a) and 1(b)). Therefore we first focused on the three amplification CNVs, which were located on 8q23.3, 13q21.1, and 18q22.1. We searched PubMed for any published functional or genome-wide studies revealing an association between the region of the three CNVs and lung cancer (Table 2). For 8q23.3, our results indicated 59.4 kb amplification region. Boelens et al. had reported 8q23.3 as a common CNV-related region of lung cancer [25]. We obtained 30.4 kb amplification on 13q21.1, a candidate region containing alterations in esophageal squamous cell carcinoma [26] and also reported by Boelens et al. [25]. We did not get direct evidence from the literature to support the third, a 9.3 kb CNV located on 18q22.1.

We also searched the literature for evidence of the 18 deletions and 1 abnormal variation recovered in our analysis. Only the 12.9 kb deletion on 5q35.2 did not appear in previous studies. Direct or indirect support was found for all other cases (Table 2), although the association consistency between EAGLE and PLCO was better at predicting amplification in EAGLE.

In further support of our CNV findings, we expected that other types of mutations, such as single-nucleotide changes, could validate the physiological significance of our predicted CNVs. We examined the mutation status of neighboring genes around these risk loci (Table S2) in the Cancer Genome Project Data of Wellcome Trust Sanger Institute (http://www.sanger.ac.uk/genetics/CGP/cosmic/).* CSMD3* in 8q23.3 is a large gene encoding a protein with CUB and sushi multiple domains and is associated with somatic mutations in lung cancer (7 mutated in 11 unique samples). Mutations in* CSMD3* were also found to be associated with familial colorectal cancer [27].* CCDC102B *in 18q22.1 is associated with somatic mutations in lung cancer (1 mutated sample). Moreover,* CSMD3* and* CCDC102B* have been reported to be affected by genomic rearrangement events in autistic patients [28] and in patients with diaphragmatic defects [29], respectively. Given the fact that these two genes in our neighboring gene list were reported with mutations in lung cancer, we expected other genes to be investigated in future studies.

### 2.5. The Risk Loci Are Located on Genomic Recombination Hotspots

Interestingly, we found that our predicted risk loci were associated with high rates of recombination (Figures 1(c) and 1(d)) compared to other SNP sites. Then we plotted the summarized 22 CNVs against HapMap hotspots on the genome (Figure S4). 13 out of 18 deletion CNVs overlapped with hotspots; 2 out of 3 amplification CNVs overlapped with hotspots; the single abnormal CNV also overlapped with hotspots. Those results revealed that there might be some connections between genomic recombination hotspots and disease risk loci; further studies are necessary to support and confirm such relationships.

## 3. Discussion

**Figure 2:**
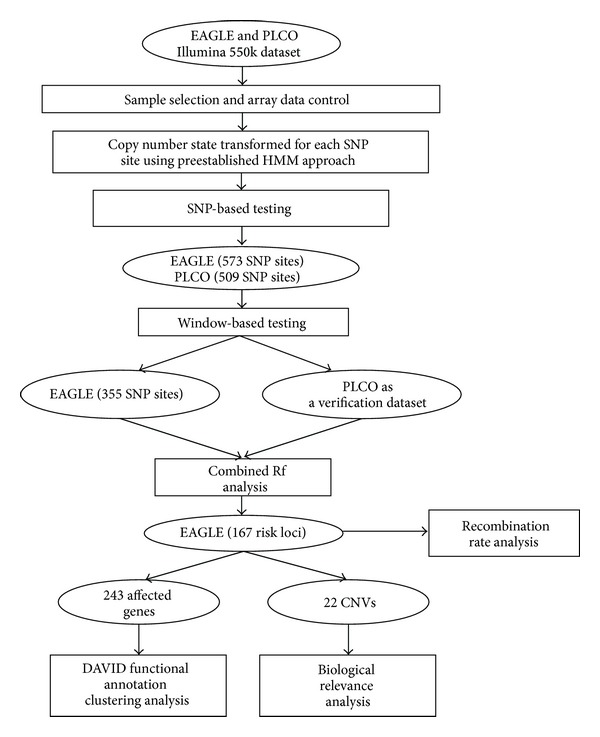
Workflow of the combined study and subsequent analysis in EAGLE.

In summary, we developed a combined analysis following our preestablished SNP-based and window-based CNV association methods to conduct a pilot study in two datasets, EAGLE and PLCO. The workflow of this pilot study can be found in Figure 2.

### 3.1. Population Structure Impact

At the study design step, we noted the population stratification in EAGLE samples (Figure S2). Although stratification only occurred within the whole population of cases and controls in EAGLE and no obvious stratification was observed between case and control cohorts, we were still concerned about whether the whole population stratification could lead to false positive results. The strategy we used to overcome this problem was to use another dataset, PLCO, as an independent verification dataset that could be integrated into our combined analysis with EAGLE. As expected, such a combined process indeed gave us meaningful results.

### 3.2. Fitting Our Results with Other GWA Studies

There was an obvious difference between our approach and other GWA studies. The majority of GWA studies performed association testing using the probe signal of an SNP site. However, our approach first transformed the original probe signal into a copy number state value using a hidden Markov model (HMM), followed by an association analysis using the transformed copy number state of an SNP site. Strictly speaking, our approach was actually a copy number state association testing. This copy number state transformation before association testing made our association risk loci unsuitable to compare directly with other GWA studies. Given this problem, we have chosen to conduct our analysis using SNP-based, window-based, and combined testing until we got a set of potentially reliable SNP risk loci. We found that there were some consecutive blocks formed in this set of risk loci (167 SNP risk loci in EAGLE, Table S2). We extracted the blocks from the set of candidate risk loci and found that these blocks were actually CNVs region predicted by our approach. We could then compare our CNVs with CNVs predicted by other studies including GWA studies.

Due to the strict three-step SNP-based, window-based, and combined analysis, when we did the literature search we were able to find direct or indirect support from previous studies for the majority of our predicted CNVs (see Table 2 for detailed validation information), which indicated the meaningfulness of our CNV predictions. We thought that, compared to other GWA studies, our approach had two advantages. First, in addition to the CNVs described by other GWA studies our approach found some new CNVs validated by other functional studies. In the popular SNP-based genome-wide association studies, some complex CNV regions might be difficult to analyze or filter by SNP site evaluation. Our approach transformed SNP signals to copy number states information, which might help our approach to maintain CNV information. Given the numerous available GWA study strategies, our approach might still give some valuable predictions that other strategies might miss. A second advantage is that our CNVs predictions did not always exactly overlap with the supporting studies' predictions. For functional evidence, our predictions might give more precise boundaries or positions of CNVs than rough functional studies because of the high-resolution array data we used. For other GWA evidence, our predictions might be a useful, complementing tool to locate CNVs more precisely.

When analyzing the risk loci, we mainly focused on CNV blocks extracted using consecutive information. However, there were many singular risk loci left (Table S2). We noted three singular risk loci among them: rs9863274 located on 3q24, rs104554013 located on 5q21.3, and rs952125 located on 21q21.1. The region 3q24 was a well-known CNV-associated locus identified in many studies of lung cancer [25, 30–32] and the amplification of this locus [30] was the most prominent difference between squamous cell carcinomas (SCCs) and adenocarcinomas (ACs) [31]. The loss of copy number in 5q21 had been previously reported to be associated with lung cancer [33] and the CNVs of this locus were implicated in clear-cell renal cell carcinoma in patients who smoked [34]. This locus might be critical in mediating interactions between environmental and genetic factors. Deletions of 21q21.1, which might correspond to a candidate tumor suppressor locus, had also been reported in lung cancer [35]. Given these CNVs supported by other studies and by our predictions, we expected these potential singular risk loci to be a set of candidate loci worthy of further functional validations.

Finally, we expected the approach developed in this study to be a valuable complement to current genome-wide association studies.

## 4. Materials and Methods

### 4.1. Data Source and Sample Selection

Our datasets were from the project “A Genome-Wide Scan of Lung Cancer and Smoking” (phs000093, the database of Genotypes and Phenotypes, dbGaP). This project consisted of two parts: (1) Environment And Genetics in Lung cancer Etiology (EAGLE) [16] and (2) the Prostate, Lung, Colon and Ovary (PLCO) Study Cancer Screening Trial [17]. These two datasets were carefully controlled for gender, age, region, and so forth. phs000093 also contained 66 individuals with European ancestry from HapMap which was used as a training dataset to estimate the parameters of the hidden Markov model (HMM).

Individuals with contamination from different genetic backgrounds and duplicated samples were filtered as per the instructions of phs000093. Finally, 1,945 cases and 1,992 controls were obtained for EAGLE and 803 cases and 848 controls were obtained for PLCO.

### 4.2. Array Data Preprocesses

The blood samples of all individuals were detected using an Illumina HumanHap 550K v3.0 genotyping chip, and these data were quantile normalized to the same baseline for further analysis. After quality control, we processed these data using SNPs annotated in the NCBI build 36 reference genome. As sex chromosomes are different from the autosomes in copy number detection and comparison, only the autosomes were studied in our work.

Finally, 547,458 autosomal SNPs annotated in NCBI build 36 reference genome were used for further analyses.

### 4.3. Population Stratification Analysis

First, we used PLINK to extract the genotype information of each SNP probe for each individual studied. Next, PLINK was used again to prune out SNPs in the 547,458 autosomal SNPs for linkage disequilibrium between SNPs with *r* ^2^ > 0.2. We then used EIGENSTRAT 3.0 software suite to do a raw smartpca analysis in EAGLE and PLCO. After the first run of smartpca, we analyzed the output snpweight of each SNP and manually removed large segments of closely flanked SNPs with abs (snpweight) > 3.5. Finally, we reran the smartpca to find top 20 significant eigenvectors in EAGLE and PLCO separately and then plotted the most significant eigenvector against the next four most significant eigenvectors for EAGLE and PLCO.

### 4.4. Copy Number State Transformation

In our analysis, the SNP probes signal data were first transformed to copy number state with a well-trained hidden Markov model (HMM). The training method and transformation process are described in our previous study [18].

### 4.5. The Simple Raw CNVs Prediction Method

In this roughly simple raw CNVs calculation step, three or more than three consecutive SNPs with the same abnormal copy number (not equal to 2) in an individual were considered to be a CNV of this sample. The description statistics in Table 1 were then calculated based on the raw CNVs generated. Note that such raw CNVs were not adopted as reliable CNVs and only used to make a comparison between EAGLE and PLCO.

### 4.6. Statistical Power Comparison between EAGLE and PLCO

Parameters for GWAPower calculation are as follows: CEU population; Illumina 550k platform; predefined *P* value of 5*e* − 7; effective size for SNPs: 1.1, 1.15, 1.2, 1.25, 1.3, 1.4, 1.5, and 1.7.2; EAGLE population size, 1945 versus 1992; and PLCO population size, 803 versus 848.

### 4.7. CNV Association Testing, Recombination Rate, and Relative Factor Calculation

CNV association testing in separate datasets was performed as a two-step statistical testing.* SNP-based testing* was performed to measure the disease association with a specific SNP site and* window-based testing* was performed to measure the CNV pattern differentiation in and around the selected SNP site. The details of these tests can be found in the original paper [18]. Here, the SNP site-based testing also includes* multiple trend testing* for a specific SNP site.

For recombination rate analysis, we downloaded the genome-wide recombination rate data from HapMap phase II (http://hapmap.ncbi.nlm.nih.gov/downloads/index.html.en). For each SNP site analyzed, we computed a sum of log10 (maximum recombination rate) within a 10 kb region of the SNP in order to represent the recombination rate level for this SNP and then compared this level among four groups of SNPs: not sig. (which consisted of nonsignificant SNPs), SNP-based (significant SNPs that passed SNP-based testing), window (significant SNPs that passed both SNP-based and window-based testing), and window_Rf (significant SNPs that passed SNP-based, window-based, and combined testing with PLCO) (see Figure 1(c)). Since HapMap phase II has already analyzed the recombination hotspot regions, we extracted the start and end positions of the hotspot regions and plotted them against the genome in Figure 1(d) and Figure S4.

The relative factor (Rf) was calculated in our analysis to test the association accordance on a specific SNP site between EAGLE and PLCO. The Rf was calculated from four models (M00, M01, M10, and M11) of the comparison between two datasets. In these models, we defined one of the datasets as the “Reference” dataset and the other one as the “Testing” dataset. Hence, the four models describe four comparisons between the two datasets.

 M00: the cases' distributions in the Testing dataset were the same as the cases' distributions in the Reference dataset.
 M10: the controls' distributions in the Testing dataset were the same as the cases' distributions in the Reference dataset.
 M01: the cases' distributions in the Testing dataset were the same as the controls' distributions in the Reference dataset.
 M11: the controls' distributions in the Testing dataset were the same as the controls' distributions in the Reference dataset.

Consider

(1) $$\begin{array}{c} \text{Rf}=\frac{P\left(\text{M}00\right)P\left(\text{M}11\right)}{P\left(\text{M}01\right)P\left(\text{M}10\right)}. \end{array}$$

Rf could be calculated using the formula shown above in which *P* values were calculated in the same manner as in SNP-based testing. We noted that the relative factor is a combination of the distribution accordance of both cases and controls. When the value is higher, the association patterns in each dataset are more similar. The advantage of using Rf is that it is compatible with the multiple hypotheses model of SNP-based testing, which was effective in our previous study [18].

### 4.8. Correction of Multiple Tests by Calculating False Discovery Rates (FDR)

Confirming the significance of multiple tests for a CNV association study is an important issue in genome-wide association analysis. CNV association *P* values are not independent of but tend to be related to the neighboring sites because CNVs may span thousands of nucleotides in the human genome. Classical Bonferroni correction was not adopted in our analysis but a permutation-based method was used to calculate false discovery rates (FDR) of a significant level. The FDR of* SNP-based testing* and* window-based testing* were calculated in the same way as in our previous work [18].

In the case of relative factors, the case-control labels for all individuals were permuted 100 times and the calculation of every model was according to the previous work [18]. The FDR could be calculated as follows:

(2)  FDRRf=NRf(m)≤RfsiteSNPTpm·NRf≤RfsiteSNP. 

Rf_site_ denotes a designated Rf value in the observed data, Rf and Rf^(*m*)^ denote the Rf values in the observed data and the permutated data, respectively, *N* ^SNP^ denotes the number of SNP sites, and *T*_pm_ denotes the number of permutations.

### 4.9. Predictions of CNVs Using Risk Loci in EAGLE

We predicted a set of potential reliable CNVs from 167 risk loci in EAGLE (Table S2). We manually investigated the 167 risk loci using the following standards to predict CNVs.

- CNVs should span three or more than three consecutive risk loci.
- “Consecutive” means the distance between two neighboring SNPs should not be larger than 30 kb.
- The type of CNVs depends on the related *P* value in Table S2.

### 4.10. Software Tools Used in This Study

We used PLINK 1.07 (Shaun Purcell, http://pngu.mgh.harvard.edu/purcell/plink/) [36], EIGENSTRAT [37], GWAPower [38], and DAVID [39] in this study.

## Supplementary Material

The Supplementary Material includes figures and tables that are complementary to our conclusions.

## Figures and Tables

**Table 1:** Descriptions of raw CNVs in EAGLE and PLCO.

	Average CNVs per individual	SNPs per CNV			CNV size		
		Min	Max	Average	Min (bp)	Max (Mb)	Average (kb)
EAGLE							
Case	391.1	3	1325	8.8	23	22.9	36.7
Control	441	3	693	8	23	8.6	32.7
PLCO							
Case	193.2	3	822	8.6	37	22.9	35.7
Control	106.7	3	537	7.5	45	2.2	30.6

Note that the raw CNVs in this table were roughly generated by a simple arbitrary method and might not be reliable.

**Table 2:** Predicted CNVs in EAGLE.

Dataset	Chr.	Band	Start_pos.	End_pos.	Size (kb)	Type	Literature
EAGLE	1	1p36.22	12120766	12129342	8.6	Deletion	19513508; 16142337; 20676096
EAGLE	5	5q35.2	175888783	175901659	12.9	Deletion	—
EAGLE	8	8q23.3	113681735	113741162	59.4	Amplification	19324446
EAGLE	8	8q24.3	144694717	144728743	34.0	Deletion	18990762; 22142333
EAGLE	8	8q24.3	145079175	145118650	39.5	Deletion	18990762; 22142333
EAGLE	9	9q32	114406899	114414974	8.1	Deletion	18798555; 15580306; 7512370
EAGLE	9	9q34.3	138620438	138641922	21.5	Deletion	16740712
EAGLE	10	10q22.3	80766077	80778488	12.4	Deletion	18758299; 20651054
EAGLE	11	11q13.1	65012165	65051406	39.2	Deletion	11274644
EAGLE	13	13q21.1	56772821	56803216	30.4	Amplification	20200074; 19324446
EAGLE	16	16p13.3	1951065	1994156	43.1	Deletion	17086460
EAGLE	17	17q21.1	35509120	35510616	1.5	Deletion	16733218; 11378338
EAGLE	17	17q25.3	73635123	73655682	20.6	Deletion	17086460
EAGLE	17	17q25.3	77848326	78009203	160.9	Deletion	17086460
EAGLE	18	18p11.32	2580764	2629683	48.9	Deletion	19190329
EAGLE	18	18q22.1	64897188	64906488	9.3	Amplification	—
EAGLE	19	19p13.3	1046061	1126396	80.3	Deletion	21521776
EAGLE	19	19p13.3	1994271	2001823	7.6	Deletion	21521776
EAGLE	19	19p13.3	2050820	2079054	28.2	Deletion	21521776
EAGLE	20	20q13.33	61642713	61668792	26.1	Abnormal	17304513
EAGLE	21	21q22.3	45769452	45788806	19.4	Deletion	15900585
EAGLE	22	22q13.1	37667446	37704618	37.2	Deletion	10515681; 15262437

This table reports the 22 predicted CNVs summarized from risk loci (Table S2) in EAGLE. The Literature shows the PubMed unique identifier (PMID) for previous papers that provide the risk evidence for these loci. See Section 4 for detailed information.
