# Supplementary figures and images for: Combined Analysis with Copy Number Variation Identifies Risk Loci in Lung Cancer
Source: Biomed Res Int. 2014 Jul 1;2014:469103. doi: 10.1155/2014/469103 (PMC4100386; doi:10.1155/2014/469103)

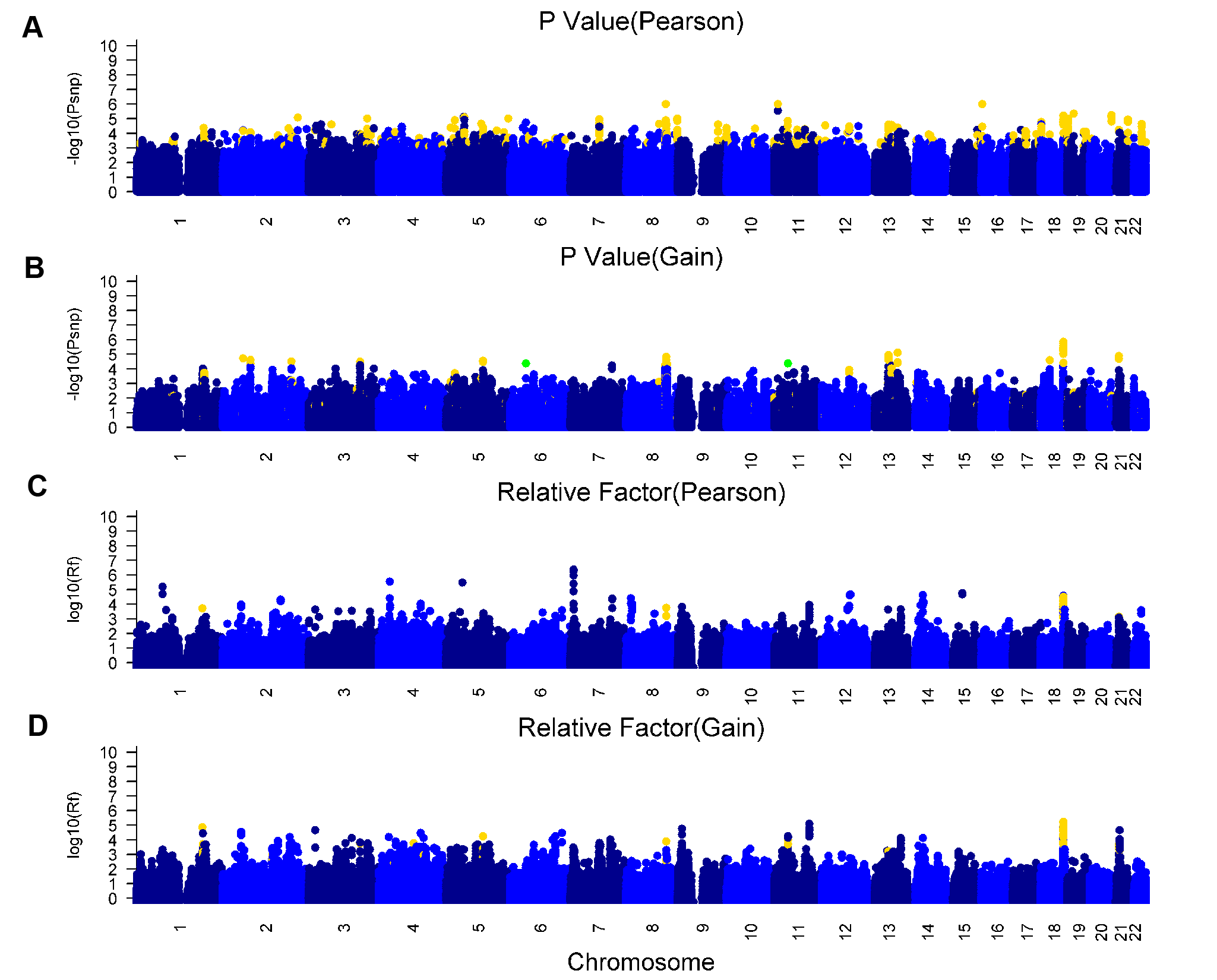

Supplement: Supplementary file 1 — The Supplementary Material includes figures and tables that are complementary to our conclusions. [file 469103.f1.zip › 469103.f1/Figure S1.tif]

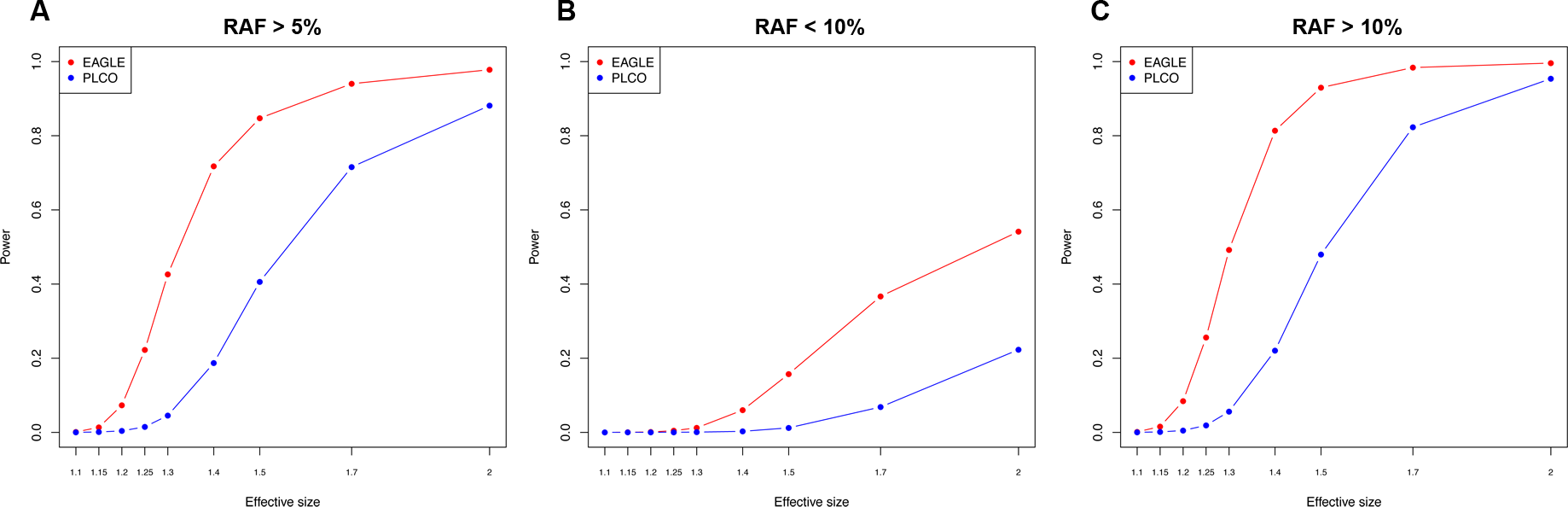

Supplement: Supplementary file 1 — The Supplementary Material includes figures and tables that are complementary to our conclusions. [file 469103.f1.zip › 469103.f1/Figure S2.tif]

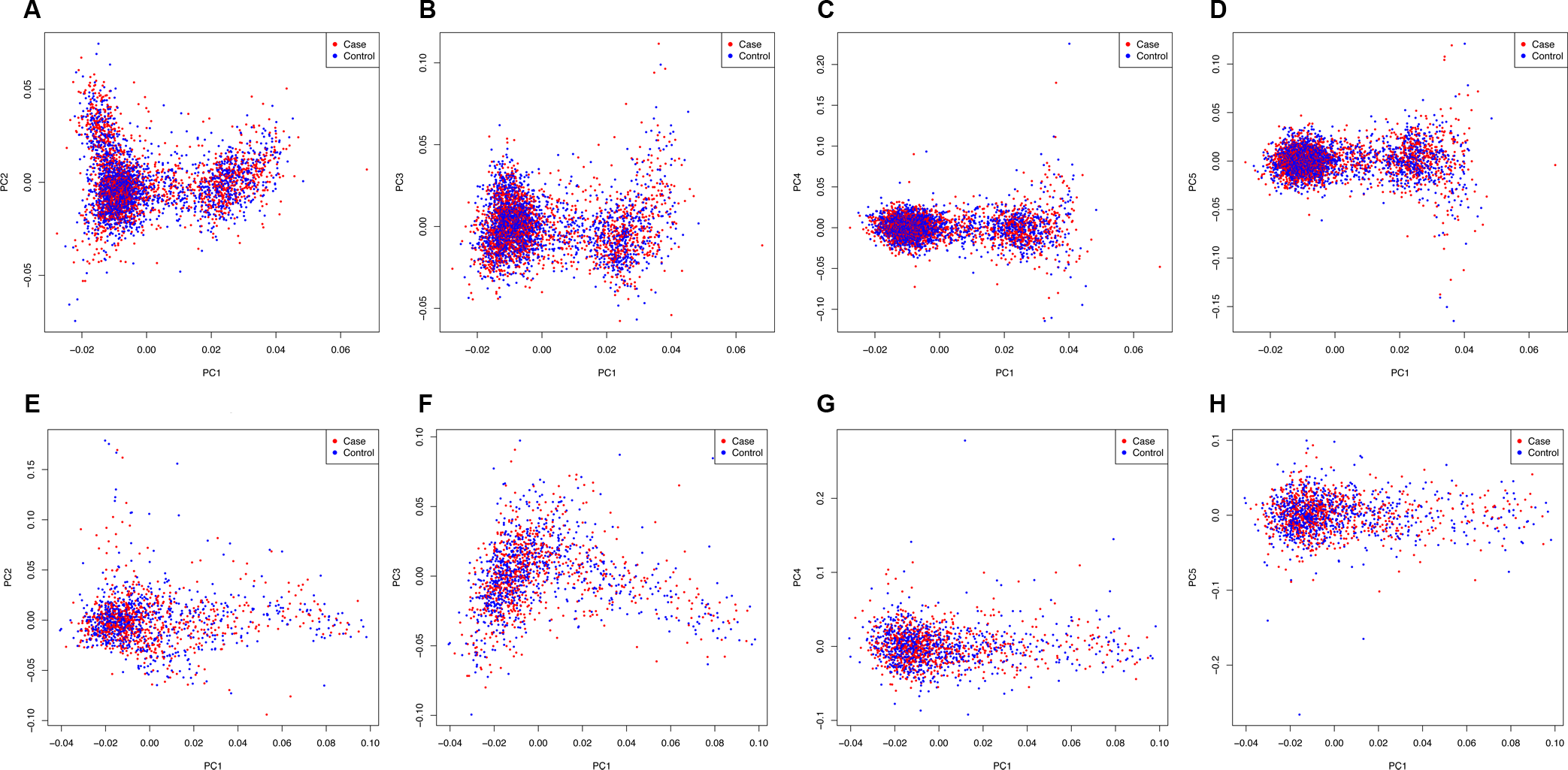

Supplement: Supplementary file 1 — The Supplementary Material includes figures and tables that are complementary to our conclusions. [file 469103.f1.zip › 469103.f1/Figure S3.tif]

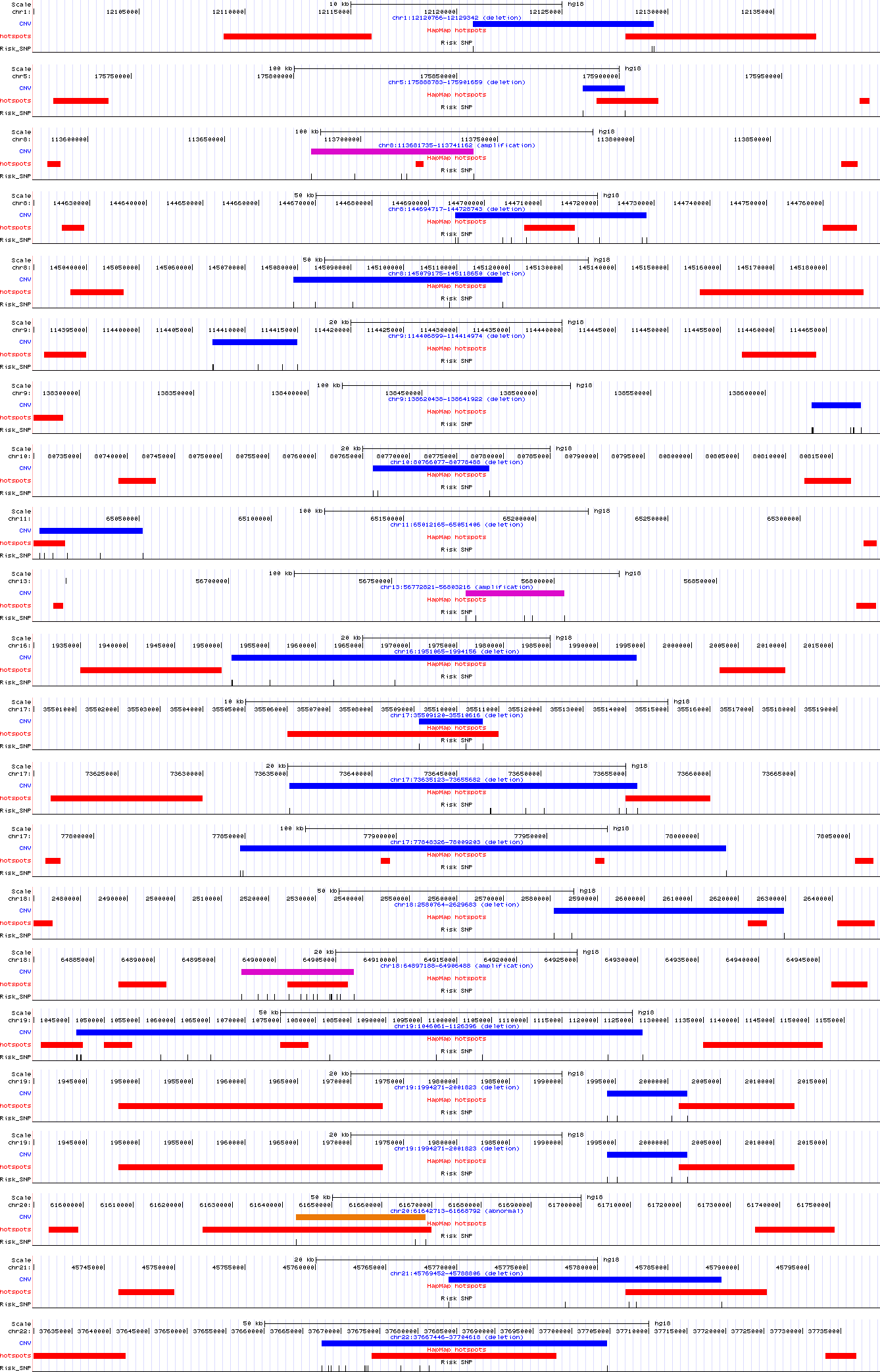

Supplement: Supplementary file 1 — The Supplementary Material includes figures and tables that are complementary to our conclusions. [file 469103.f1.zip › 469103.f1/Figure S4.tif]
